# PTTG1 Enhances Oncolytic Adenovirus 5 Entry into Pancreatic Adenocarcinoma Cells by Increasing CXADR Expression

**DOI:** 10.3390/v15051153

**Published:** 2023-05-11

**Authors:** Lu Long, Jian Gao, Ruiyang Zhang

**Affiliations:** 1 Department of Clinical Laboratory, Shanghai Ninth People’s Hospital, Shanghai Jiao Tong University School of Medicine, Shanghai 200011, China; longl199084@163.com; 2 State Key Laboratory of Oncogenes and Related Genes, Shanghai Cancer Institute, Shanghai Jiao Tong University School of Medicine, Shanghai 200032, China; jian.gao@uni-wh.de

**Keywords:** PTTG1, pancreatic adenocarcinoma, oncolytic adenovirus, CXADR

## Abstract

Pituitary tumor-transforming gene 1 (PTTG1) is overexpressed in various types of tumors and functions as an oncogene; it could also be a potential target in tumor therapy. Meanwhile, the high mortality of pancreatic adenocarcinoma (PAAD) largely depends on the limited effectiveness of therapy. Based on the promising potential of PTTG1 in cancer treatment, we explored the influence of PTTG1 on the treatment of PAAD in this study. The Cancer Genome Atlas Program (TCGA) data showed that higher expression of PTTG1 was associated with higher clinical stages and worse prognosis of pancreatic cancer. In addition, the CCK-8 assay showed that the IC50 of gemcitabine and 5-fluorouracil (5-FU) was increased in BxPC-3-PTTG1^high^ and MIA PaCa-2-PTTG1^high^ cells. The TIDE algorithm indicated that the immune checkpoint blockades’ (ICBs) efficiency is poor in the PTTG1 high group. Furthermore, we found that the efficiency of OAd5 was enhanced in BxPC-3-PTTG1^high^ and MIA PaCa-2-PTTG1^high^ cells and poor in BxPC-3-PTTG1^low^ and MIA PaCa-2-PTTG1^low^ cells. We used the OAd5 expressing GFP for transduction. As a result, the fluorescence intensity was enhanced in BxPC-3-PTTG1^high^ and MIA PaCa-2-PTTG1^high^ cells and decreased in BxPC-3-PTTG1^low^ and MIA PaCa-2-PTTG1^low^ cells 24 h after OAd5 transduction. The fluorescence intensity indicated that PTTG1 increased OAd5 entry. The flow cytometry assay showed that OAd5 receptor CXADR expression was enhanced by PTTG1. PTTG1 failed to further enhance OAd5 transduction in the case of CXADR knockdown. In summary, PTTG1 enhanced OAd5 transduction into pancreatic cancer cells by increasing CXADR expression on the cell surface.

## 1. Introduction

Pituitary tumor-transforming gene 1 (PTTG1) regulates sister chromatid separation during mitosis [1]. It also involves several cellular processes, such as DNA damage repair, apoptosis, and metabolism [2]. Furthermore, PTTG1 is overexpressed and oncogenic in many kinds of tumors, such as prostate, breast, liver, lung, and seminoma [3,4,5,6,7]. PTTG1 promotes tumor cell proliferation, invasiveness, epithelial–mesenchymal transition, and angiogenesis [8,9,10]. Given the critical tumor-promoting role of PTTG1 in tumor progression, it is necessary to comprehensively understand its potential in therapy. 

Pancreatic adenocarcinoma (PAAD) has exceptionally high mortality, with a median survival time of six–nine months and a five-year survival rate of less than 10% due to late diagnosis and limited effectiveness of treatment strategies [11,12]. Chemotherapy is mainly used in locally advanced and in most borderline resectable tumors. However, chemotherapy may induce tumor downstaging for some patients and convert unresectable to resectable disease. Presently, the acceptable first-line therapies for pancreatic cancer are 5-fluorouracil [5-FU]-based FOLFIRONOX and gemcitabine plus albumin-bound paclitaxel. However, due to chemoresistance, a significant proportion of patients with pancreatic cancer present a poor prognosis [13].

The term ‘immune checkpoint’ refers to molecules expressed on the surface of immune cells that can be used to regulate cellular immune responses [14]. Tumor cells cause the exhaustion and functional inhibition of anti-tumor immune cells through immune checkpoints, thereby avoiding the killing effect of immune responses and achieving immune escape. The basic principle of immune checkpoint blockades (ICBs) is to ease the inhibition of immune checkpoints on immune cells and utilize the endogenous anti-tumor response of the immune system to fight against diseases [14]. The frequently studied immune checkpoints are cytotoxic T lymphocyte protein 4 (CTLA-4), programmed death 1 (PD-1), and programmed death ligand 1 (PD-L1). CTLA-4 binding with ligands inhibits the activation of T lymphocytes, so blocking CTLA-4 could increase T cell activation. PD-1 is expressed on T and NK lymphocytes. PD-L1 is its primary ligand and can be produced by various cells such as tumor cells, neutrophils, and macrophages. PD-1 binds to PD-L1, and PD-1 transmits inhibitory signals within immune cells. Therefore, blocking PD-1/PD-L1 binding can enhance T cell activation and tumor immune response [15]. Immune checkpoint molecules are critical modulators of initiating and terminating antitumor immune response [16]. Immune checkpoint blockades have been proven successful in multiple cancers [17]. However, ICBs using anti-CTLA-4 (NCT01473940, NCT02527434, NCT02558894, NCT02879318) and anti-PD-1/PD-L1 (NCT01876511, NCT03214250, NCT02866383, NCT02323191, NCT03637491) in clinical trials did not show efficacy in pancreatic cancer due to the immunosuppressive tumor microenvironment of PAAD and multiple factors inducing resistance [18,19,20]. 

Therefore, alternative therapies for PAAD are much needed to increase clinical benefits. Oncolytic viruses target and destroy cancer cells while minimizing the toxicity to normal cells. Currently, tumor immunotherapies have attracted increasing attention in the treatment of advanced cancer. Among them, oncolytic viruses have attracted extensive attention because of their high oncolytic effect and low drug resistance rate. Oncolytic viruses are natural or engineered viruses that can specifically recognize and infect tumor cells and selectively reproduce in tumor cells, thus directly inducing the lysis of malignant cells, but not damaging normal cells [21]. After killing tumor cells, oncolytic viruses release offspring ones, which infect nearby tumor cells again and repeat this process, thereby killing a larger range of tumor cells [22]. Adenovirus is a common pathogen that can infect multiple organs in the human body, but the clinical symptoms are quite mild [23]. Virus DNA replication and capsid assembly take place in the nucleus, and the infected cells split to release virus progeny. In addition, lysed tumor cells can also release new tumor antigens, viral pathogen-associated molecular patterns (PAMP), and damage associated molecular patterns to promote anti-tumor immune responses. Since adenoviruses can infect a large number of epithelial cells, they are preferably used to construct oncolytic viruses [24]. Oncolytic adenoviruses, especially oncolytic Ad serotype 5 (OAd5), are emerging choices for cancer treatments [25]. Adenoviruses are small non-enveloped viruses with a 30–38 kb linear double-stranded genome encapsidated in an icosahedral capsid [26]. OAd5 infects cells via the binding of viral fiber to the coxsackie virus and adenovirus receptor (CXADR) on the epithelial cell surface. These OAd5 have proven promising efficacy and tumor-selectivity in preclinical pancreatic cancer research. 

However, for cancer cells that poorly express CXADR, the oncolysis efficiency of OAd5 is also very low. Therefore, understanding the chemotherapy, immunotherapy, and OV resistance mechanism is significant for exploring innovative therapy alternatives. 

This study found that PTTG1 expression was increased in pancreatic cancer tumor tissues and was associated with poor prognosis. The half maximal inhibitory concentration (IC50) of gemcitabine and 5-fluorouracil (5-FU) was raised in PTTG1-overexpressed pancreatic cells. The TIDE algorithm indicated that the ICBs’ efficiency is poor in the high PTTG1 group. The efficiency of OAd5 was enhanced in PTTG1-overexpressed pancreatic cells. PTTG1 increased OAd5 entrance, but not replication, in cells. In addition, the expression of OAd5 receptor CXADR was enhanced in PTTG1-overexpressed pancreatic cells. PTTG1 failed to further enhance OAd5 transduction in the case of CXADR knockdown. To our knowledge, this is the first study showing that PTTG1 enhanced OAd5 transduction into cancer cells, and it has crucial implications for optimized OAd5 therapy.

## 2. Materials and Methods

### 2.1. Data Collection and Analysis of PTTG1 Expression

PTTG1 expression profiles and clinical information of pancreatic cancer are from the Cancer Genome Atlas Program (TCGA) database (https://portal.gdc.com). For PTTG1 expression analysis in tumor and normal tissues, we used GEPIA 2.0 (http://gepia2.cancer-pku.cn/#index) to analyze 179 tumor tissues and 171 normal tissues. 

We used the ACLBI database (https://www.aclbi.com/static/index.html#/) to analyze the relationship between PTTG1 and WHO stages and survival. For PTTG1 expression analysis in different WHO stages, we selected a total of 179 patients with completed stage information. Statistical analysis and ggplot2 (v3.3.2) were completed using R program v4.0.3; *p* < 0.05 was considered statistically significant. There was a distribution of clinical characteristics in the samples from different groups. The abscissa represents samples from different groups, and the ordinate represents the percentage of clinical sample information in corresponding groups; different colors represent different clinical information. The figure below represents the distribution of a clinical feature between two arbitrary groups, and the significance *p* value was analyzed via chi-square test, where the value is displayed as −log10 (*p* value). Marked with * indicates a significant difference in the distribution of the clinical features between the two groups (*p* < 0.05).

According to the ACLBI database, 179 patients with overall survival, disease-specific survival, and progression-free interval information were selected, respectively. Kaplan–Meier survival analysis of the gene signature from the TCGA dataset and comparison among different groups was made using log-rank test. For Kaplan–Meier curves, *p*-values and hazard ratios (HRs) with 95% confidence interval (CI) were generated using log-rank tests and univariate Cox proportional hazards regression. All the analysis methods and R packages were implemented with R (foundation for statistical computing 2020) version 4.0.3. All patients were divided into two groups according to PTTG1 expression media. HR represents the hazard ratio of the low-expression sample relative to the high-expression sample. *p* value < 0.05 was considered statistically significant. 

### 2.2. Immune Checkpoints’ Blockade Response 

We used the ACLBI database (https://www.aclbi.com/static/index.html#/) to analyze the immune checkpoints’ blockade response. RNA-sequencing expression (level 3) profiles and corresponding clinical information for pancreatic cancer were downloaded from the TCGA dataset. Potential ICB responses were predicted with TIDE algorithm. TIDE uses a set of gene expression markers to evaluate two different mechanisms of tumor immune escape, including dysfunction of tumor-infiltrating cytotoxic T lymphocytes (CTLs) and rejection of CTLs by immunosuppressive factors. TIDE score is high, ICBs have poor efficacy, and survival after receiving ICB treatment is short.

### 2.3. Cell Culture

The PAAD cell BxPC-3 was cultured in RPMI-1640 Medium (Gibco) supplemented with 10% fetal bovine serum (FBS; BIOSUN) and 100 U/mL penicillin/streptomycin (Hyclone). The pancreatic carcinoma cell MIA PaCa-2 was cultured in Dulbecco’s Modified Eagle’s Medium (DMEM, Gibco) supplemented with 10% FBS, 2.5% horse serum, and 100 U/mL penicillin/streptomycin. The cells were cultured in 37 °C and 5% CO_2_ incubators.

### 2.4. Stable Cell Line Construction

Total RNA was extracted from BxPC-3 cells, then was reverse transcribed into cDNA. The cDNA was used as a template for PCR amplification to obtain the coding region of PTTG1. The primers used for PCR reactions are as follows: forward primer, 5′-CTAGCTAGCatggctactctgatctatgttgat-3′; reverse primer, 5′-CGCGGATCCttaaatatctatgtcacagcaaacag-3′. The capital letters CTA at the start of forward primer and the capital letters CGC at the start of reverse primer are the addition of terminal bases to reduce asymmetric cleavage. The NheI-HF restriction sites are underlined in forward primer, the BamHI-HF restriction sites are underlined in reverse primer. The lower case letters are sequences that bind to the coding region of PTTG1. The amplified coding regions were inserted into the pJET1.2/blunt vector (ThermoFisher Scientific, Waltham, MA, USA), obtaining the pJET1.2-PTTG1 vectors. Then, the PTTG1 coding region was transferred from the pJET1.2-PTTG1 to the plasmid pCMV-IRES-Neo/KanR using the Restriction Enzyme Digestion and Ligation protocol. NheI-HF and BamHI-HF were used for digesting JET1.2-PTTG1 vector, and T4 ligase was used for ligating PTTG1 coding regions into pCMV-IRES-Neo/KanR vector. Then, we obtained the PTTG1 overexpression plasmids. For constructing BxPC-3-PTTG1^high^ and MIA PaCa-2-PTTG1^high^ cells, the PTTG1 overexpression plasmids were transfected into BxPC-3 and MIA PaCa-2 cells using LipofectamineTM 3000 (Life Technologies Corp, Carlsbad, CA, USA). 

For constructing BxPC-3-PTTG1^low^ and MIA PaCa-2-PTTG1^low^ cells, the guide RNA (gRNA) for knocking PTTG1 using CRISPR/Cas9 was designed based on the genome GRCH38 (hg38, Homo sapiens) on the website https://www.benchling.com/crispr/. The gRNA targets the exon 1 of PTTG1, and its sequence was 5′-CCCATCCTTAGCAACCACAC-3′. The CRISPR/Cas9 backbone was a gift from Feng Zhang through Addgene. The gRNA was inserted in to the CRISPR/Cas9 backbone using BbsI-HF (NEB); the process was according to a previous report [27]. The CRISPR/Cas9 vector was transfected into BxPC-3 and MIA PaCa-2 cells.

Transfected cells were selected using 600 µg /mL G418 (Life Technologies Corp, Hong Kong, China) for BxPC-3-PTTG1^high^ and BxPC-3-PTTG1^low^ cells, and 500 µg /mL G418 for MIA PaCa-2-PTTG1^high^ and MIA PaCa-2-PTTG1^low^ cells. The G418 selection lasted for two weeks, and the single-cell clones were amplified and verified using Western Blot Analysis and flow cytometry assay. The stable cells were maintained in 250 µg /mL G418. 

### 2.5. Western Blot Analysis

For the detection of PTTG1 expression in BxPC-3 and MIA PaCa-2 cells, we prepared BxPC-3-PTTG1^high^, MIA PaCa-2-PTTG1^high^, BxPC-3-PTTG1^low^, and MIA PaCa-2-PTTG1^low^ cells. Whole-cell lysates were prepared using RIPA buffer (Thermofisher Scientific), and 100 μg of total protein was run in 10% SDS-polyacrylamide gel and transferred onto polyvinylidene fluoride (PVDF) membrane (Invitrogen, Waltham, MA, USA). The membrane was blocked using 5% milk/Tris-buffered saline plus Tween 20 (TBST), followed by incubation with the primary antibody against PTTG1 (Thermofisher Scientific). After washing with the TBST, the membrane was incubated with HRP-conjugated mouse anti-human IgG secondary antibody conjugated to horseradish peroxidase. α-tubulin was used as the internal control. 

### 2.6. CCK-8 Assay

Cell viability was assessed using CCK-8 assay (Sigma-Aldrich, St. Louis, MO, USA), and it was conducted following manuscripts’ instruction. For the detection of IC50 of gemcitabine and 5-FU, BxPC-3-PTTG1^high^, MIA PaCa-2-PTTG1^high^, BxPC-3-PTTG1^low^, and MIA PaCa-2-PTTG1^low^ cells in 24-well plates were incubated with various concentrations (0, 0.5, 1, 2, 5, 10, 20 nM) of gemcitabine and 5-FU for 72 h and then further incubated with CCK-8 for 4 h. 

E1B55K- and E3B-deleted OAd5 were purchased from the GeneChem company (Shanghai, China). For the detection of cell viability after OAd5 transduction, BxPC-3-PTTG1^high^, MIA PaCa-2-PTTG1^high^, BxPC-3-PTTG1^low^, and MIA PaCa-2-PTTG1^low^ cells in 96-well plates were transduced with OAd5 in 10 vp/cell. Before CCK-8 assay, thaw the CCK-8 in a water bath at 37 °C. Afterwards, add 10 μL of the CCK-8 reagent to each well of the plate at 0, 24, 48, and 72 h after transduction. Incubate the plate for 2 h in the incubator. CCK-8 assay was performed at an absorbance of 450 nm with a microplate reader (Tecan, Männedorf, Switzerland). All experiments were performed three times.

### 2.7. Fluorescence Visualization and Quantification

24 h post OAd5 transduction, the visualization images were taken at GFP channel using a Zeiss microscope (Axio Observer 7). 0, 24, 48, and 72 h after transduction, the fluorescence intensity was measured using a Synergy2 Multi-Mode Microplate Reader (BioTek, Hong Kong, China).

### 2.8. Flow Cytometry Assay

After transduction with OAd5, the fluorescence was monitored in FITC channel with a flow cytometry assay (BD FACSCelesta). BxPC-3-PTTG1^high^, MIA PaCa-2-PTTG1^high^, BxPC-3-PTTG1^low^, and MIA PaCa-2-PTTG1^low^ cells were stained with FITC rabbit anti-human CXADR (clone no. 271, Invitrogen) for 1 h at room temperature. After washing with PBS 3 times, the expressions were measured with a flow cytometry assay.

### 2.9. Data Collection and Analysis of CXADR Expression

CXADR expression profiles and clinical information are from the TCGA pancreatic cancer database. For CXADR expression analysis in tumor and normal tissues, we used GEPIA 2.0 to analyze 179 tumor tissues and 171 normal tissues. We used the GEPIA 2.0 website to analyze the Spearman correlation between PTTG1 and CXADR mRNA expressions based on TCGA Tumor, TCGA Normal, and GTEx databases. 

According to the ACLBI database (https://www.aclbi.com/static/index.html#/), 179 patients with overall survival and disease-specific survival were selected for Kaplan–Meier survival analysis. All patients were divided into two groups according to CXADR expression media. All the analysis methods and R packages were implemented using R (foundation for statistical computing 2020) version 4.0.3. *p* < 0.05 was considered statistically significant.

### 2.10. Statistical Analysis

Data are presented as the mean ± standard deviation (SD). Student’s *t*-test (two-tailed) was used to analyze differences between two groups using R software (version: 3.6.2). Nonparametric test was used to analyze differences between two groups whose data did not conform to the normal distribution. Spearman’s rank correlation coefficient was used to analyze the correlation between two factors. *p* < 0.05 was considered statistically significant: * *p* < 0.05, ** *p* < 0.01, and *** *p* < 0.001. All data participating in statistical analysis, *n* ≥ 3.

## 3. Results

### 3.1. High PTTG1 Expression Correlates with High Clinical Stage and Poor Pancreatic Cancer Prognosis

**Figure 1:**
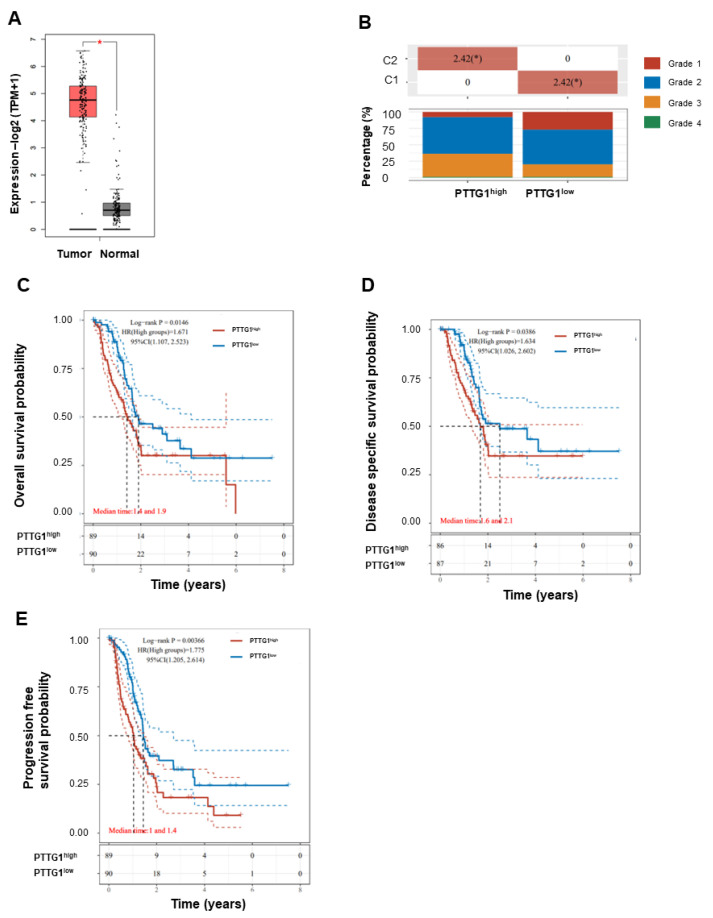
The correlation between PTTG1 and Pancreatic Cancer Prognosis. (**A**) PTTG1 expression analysis in PAADa from TCGA database using GEPIA 2.0. 179 cases of pancreatic cancer tumor tissues and 171 cases of normal pancreas tissues were used. (**B**) The association between PTTG1 expression and the clinical stage was analyzed in the TCGA cohort. The association between (**C**) PTTG1 expression and overall survival, (**D**) PTTG1 expression and disease-specific survival, and (**E**) PTTG1 expression and progression-free survival were analyzed in the TCGA cohort. Hazard ratio (HR) > 1 indicates the gene is a risk factor. HR (95% Cl), the median survival time (LT50) for different groups. *: *p* < 0.05.

We first assessed PTTG1 expression in pancreatic cancer from the TCGA database. The analysis revealed that PTTG1 expression was higher in pancreatic cancer tumor tissues than in normal ones (Figure 1A). Furthermore, the expression of PTTG1 was closely related to the clinical stage, being higher in pancreatic cancer patients with relatively high stages (Figure 1B). To evaluate the value of PTTG1 in predicting the prognosis of pancreatic cancer patients, we analyzed the association between its expression and overall survival (OS), disease-free survival (DFS), progression-free survival (PFS), and disease-specific survival (DSS) in the TCGA cohort. Higher expression of PTTG1 was significantly associated with shorter OS (Figure 1C) and DSS (Figure 1D) in pancreatic cancer. In addition, higher expression of PTTG1 was also significantly associated with a reduction in PFS (Figure 1E) in pancreatic cancer. The analysis indicates that PTTG1 is involved in disease progression in pancreatic cancer.

### 3.2. Increased PTTG1 Expression Correlates with Chemotherapy and Immunotherapy Resistance

**Figure 2:**
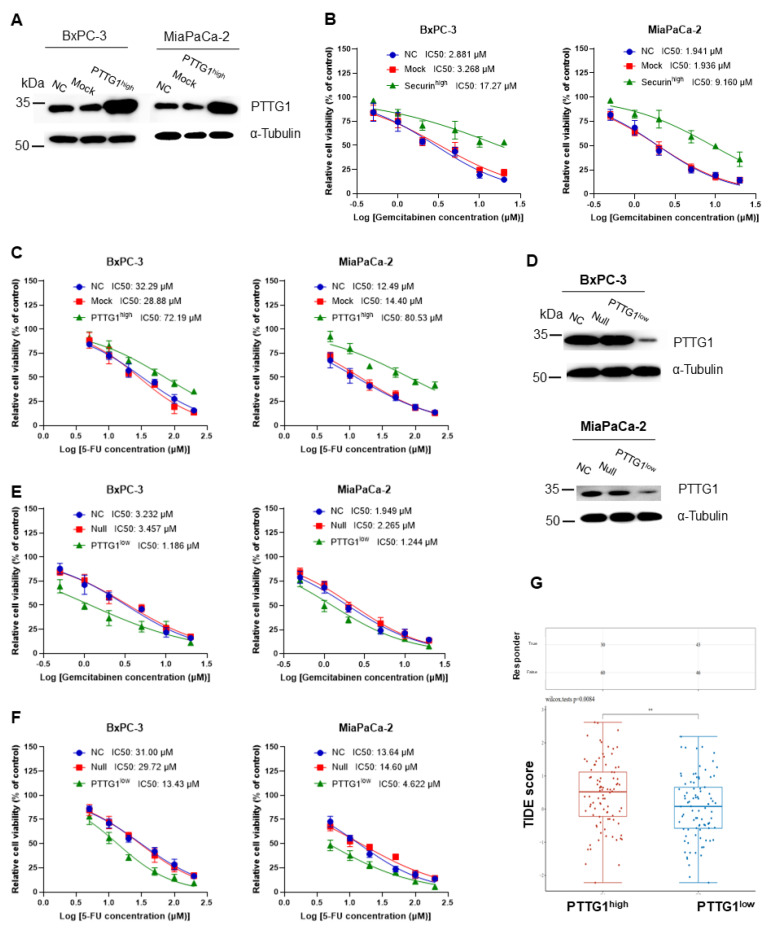
PTTG1 increased chemotherapy and immunotherapy resistance in pancreatic cancer cells. (**A**) Overexpressed PTTG1 in BxPC-3 and MIA PaCa-2 cells. The IC50 of (**B**) gemcitabine and (**C**) 5-fluorouracil (5-FU) in PTTG1-overexpressed BxPC-3 (BxPC-3-PTTG1^high^) and MIA PaCa-2 (MIA PaCa-2-PTTG1^high^) cells. (**D**) Knocked down PTTG1 in BxPC-3 and MIA PaCa-2 cells using CRISPR/Cas9. The IC50 of (**E**) gemcitabine and (**F**) 5-fluorouracil (5-FU) in PTTG1 knockdown BxPC-3 (BxPC-3-PTTG1^low^) and MIA PaCa-2 (MIA PaCa-2-PTTG1^low^) cells. Cell viability was measured using CCK-8 assay. All IC50 experiments were conducted in triplicate. (**G**) The ICB response with TIDE algorithm in PTTG1^high^ and PTTG1^low^ groups of pancreatic cancer. NC: parental cells without treatment. Mock: cells transfected with pCMV-IRES-Neo/KanR backbone vectors. PTTG1^high^: cells overexpressing PTTG1. Null: cells transfected with CRISPR/Cas9 backbone vectors. PTTG1^low^: cells knocking PTTG1. **: *p* < 0.01.

Chemotherapy is among the primary therapies for pancreatic cancer, and gemcitabine and 5-fluorouracil (5-FU) are the primary chemotherapy medicines. To further explore the influence of PTTG1 on chemotherapy, we assessed the IC50 of gemcitabine and 5-FU in PTTG1 overexpression and knockdown cells. First, we overexpressed PTTG1 in BxPC-3 and MIA PaCa-2 cells (Figure 2A). The IC50 of gemcitabine (Figure 2B) and 5-fluorouracil (5-FU) (Figure 2C) increased in PTTG1-overexpressed BxPC-3 (BxPC-3-PTTG1^high^) and MIA PaCa-2 (MIA PaCa-2-PTTG1^high^) cells. Then, we knocked PTTG1 in BxPC-3 and MIA PaCa-2 cells (Figure 2D). Correspondingly, the IC50 of gemcitabine (Figure 2E) and 5-fluorouracil (5-FU) (Figure 2F) decreased in PTTG1 knockdown BxPC-3 (BxPC-3-PTTG1^low^) and MIA PaCa-2 (MIA PaCa-2-PTTG1^low^) cells. Furthermore, we predicted potential ICB response with the TIDE algorithm in pancreatic cancer, finding that the ICBs’ efficiency is poor in the PTTG1 high group (Figure 2G). The results indicate that chemotherapy and ICBs are not suitable remedies in pancreatic cancer patients with high PTTG1 expression, and we should explore alternatives for treating PAAD.

### 3.3. PTTG1 Enhanced Oncolytic Adenovirus Efficiency in Pancreatic Cancer Cells

**Figure 3:**
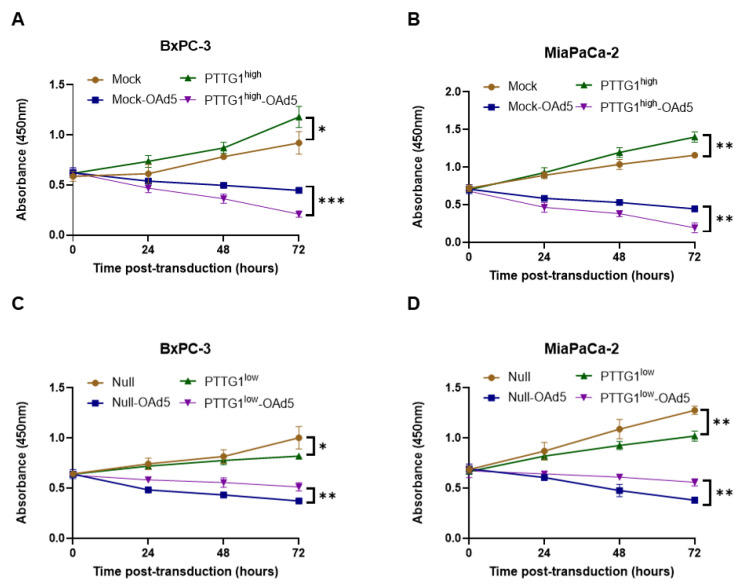
Influence of PTTG1 on OAd5 efficiency on pancreatic cancer cells. (**A**) BxPC-3-PTTG1^high^, (**B**) MIA PaCa-2-PTTG1^high^, (**C**) BxPC-3-PTTG1^low^, and (**D**) MIA PaCa-2-PTTG1^low^ cells were transduced with OAd5, cell viability was monitored using CCK-8 assay 0-, 24-, 48- and 72-h post-transduction. Mock: cells transfected with pCMV-IRES-Neo/KanR backbone vectors. PTTG1^high^: cells-overexpressing PTTG1. Mock-OAd5: cells carrying pCMV-IRES-Neo/KanR backbone were transduced with OAd5. PTTG1^high^-OAd5: cells overexpressing PTTG1 were transduced with OAd5. Null: cells transfected with CRISPR/Cas9 backbone vectors. PTTG1^low^: cells knocking PTTG1. Null -OAd5: cells carrying CRISPR/Cas9 backbone were transduced with OAd5. PTTG1^low^-OAd5: cells knocking PTTG1 were transduced with OAd5. All experiments were conducted in triplicate. Nonparametric test was used to analyze differences between two groups. All data are mean ± SD. *: *p* < 0.05. **: *p* < 0.01. ***: *p* < 0.001.

Due to the potential of oncolytic viruses in cancer treatment, we tried to further explore the impact of PTTG1 on the efficiency of OAd5 in killing pancreatic cancer cells. We first constructed BxPC-3-PTTG1^high^ and MIA PaCa-2-PTTG1^high^ cells stably overexpressing human PTTG1, with their negative control cells (Mock) stably carrying the backbone vector. We transduced BxPC-3-PTTG1^high^ and MIA PaCa-2-PTTG1^high^ cells with the OAd5 in 10 vp/cell, then detected cell viability using a CCK-8 assay within 3 days post-transduction. Interestingly, we found that BxPC-3-PTTG1^high^ (Figure 3A) and MIA PaCa-2-PTTG1^high^ (Figure 3B) cell viability were increased compared with the relative Mock cells. Notably, the viability in BxPC-3-PTTG1^high^ (PTTG1^high^-OAd5) and MIA PaCa-2-PTTG1^high^ (PTTG1^high^-OAd5) cells was significantly decreased compared with the relative Mock cells (Mock-OAd5), indicating that the efficiency of OAd5 was enhanced by PTTG1 overexpression in BxPC-3 and MIA PaCa-2 cells.

To further validate the influence of PTTG1 on OAd5 efficiency, we knocked PTTG1 in BxPC-3 (BxPC-3-PTTG1^low^) and MIA PaCa-2 (MIA PaCa-2-PTTG1^low^) cells using CRISPR/Cas9 technology. We transduced BxPC-3-PTTG1^low^ and MIA PaCa-2-PTTG1low cells with OAd5 in 10 vp/cell. Consistently with the results from PTTG1 overexpression cells, PTTG1 knockdown decreased cell viability compared with cells carrying CRISPR/Cas9 backbone vectors (Null). Importantly, BxPC-3-PTTG1^low^ (Figure 3C) and MIA PaCa-2-PTTG1^low^ (Figure 3D) cell viability capacity were significantly decreased compared with the relative Null cells. The abovementioned results indicate that PTTG1 overexpression enhanced OAd5 efficiency, while PTTG1 knockdown reduced OAd5 efficiency. However, the underlying mechanism is unclear and needs further exploration. 

### 3.4. PTTG1 Increased the Entry of Oncolytic Adenovirus into Pancreatic Cancer Cells

**Figure 4:**
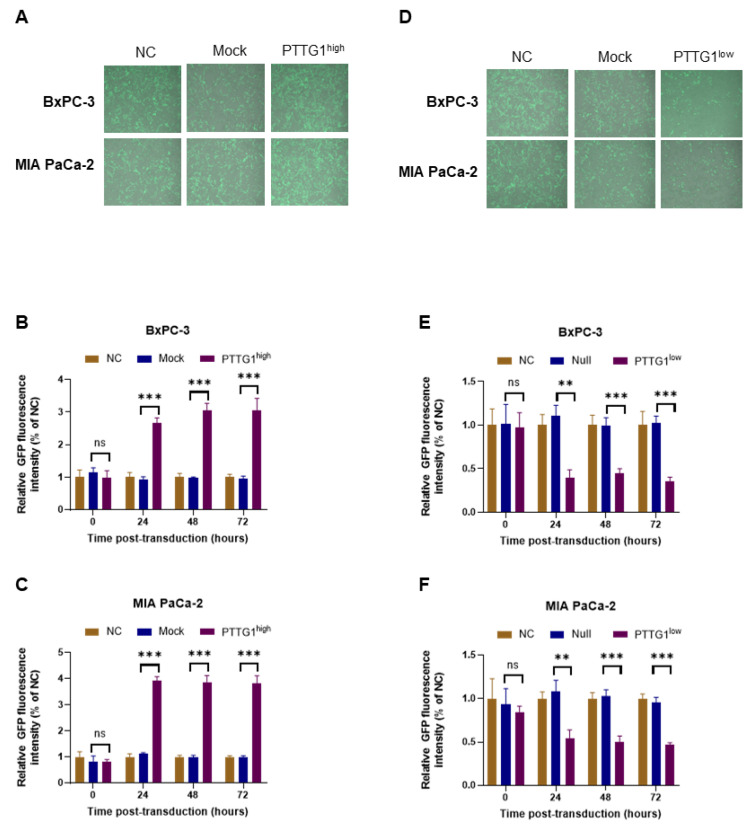
PTTG1 Increased OAd5 entry into pancreatic cancer cells. (**A**) BxPC-3 and MIA PaCa-2 cells overexpressing PTTG1 were transduced with OAd5 24 h post-transduction; GFP was detected using fluorescence microscope. Each image displayed is a merged bright field and fluorescent image. Fluorescence intensity in (**B**) BxPC-3 and (**C**) MIA PaCa-2 cells was measured 0-, 24-, 48- and 72-h post-transduction. (**D**) BxPC-3 and MIA PaCa-2 cells with PTTG1 knockdown were transduced with OAd5 24 h post-transduction; GFP was detected using a fluorescence microscope. Fluorescence intensity in (**E**) BxPC-3 and (**F**) MIA PaCa-2 cells with PTTG1 knockdown was measured 0-, 24-, 48- and 72-h post-transduction. All experiments were conducted in triplicate. Nonparametric test was used to analyze differences between two groups. All data are mean ± SD. ns: not significant. **: *p* < 0.01. ***: *p* < 0.001.

We then measured GFP expression in BxPC-3 and MIA PaCa-2 cells after OAd5 transduction. The results showed that the fluorescence intensity was enhanced in BxPC-3-PTTG1^high^ and MIA PaCa-2-PTTG1^high^ cells (Figure 4A) and decreased in BxPC-3-PTTG1^low^ and MIA PaCa-2-PTTG1^low^ cells (Figure 4D) 24 h after OAd5 transduction. However, the magnitude of the difference in GFP intensity from the control group did not further increase in (Figure 4B) BxPC-3-PTTG1^high^ and (Figure 4C) MIA PaCa-2-PTTG1^high^ cells or decrease in (Figure 4E) BxPC-3-PTTG1^low^ and (Figure 4F) MIA PaCa-2-PTTG1^low^ over time. Because OAd5 mainly replicates 24–48 h after transduction, the results indicate that PTTG1 might increase OAd5 entrance into pancreatic cancer cells, but it does not play a role in its replication.

### 3.5. PTTG1 Increased CXADR Expression on Pancreatic Cancer Cells

**Figure 5:**
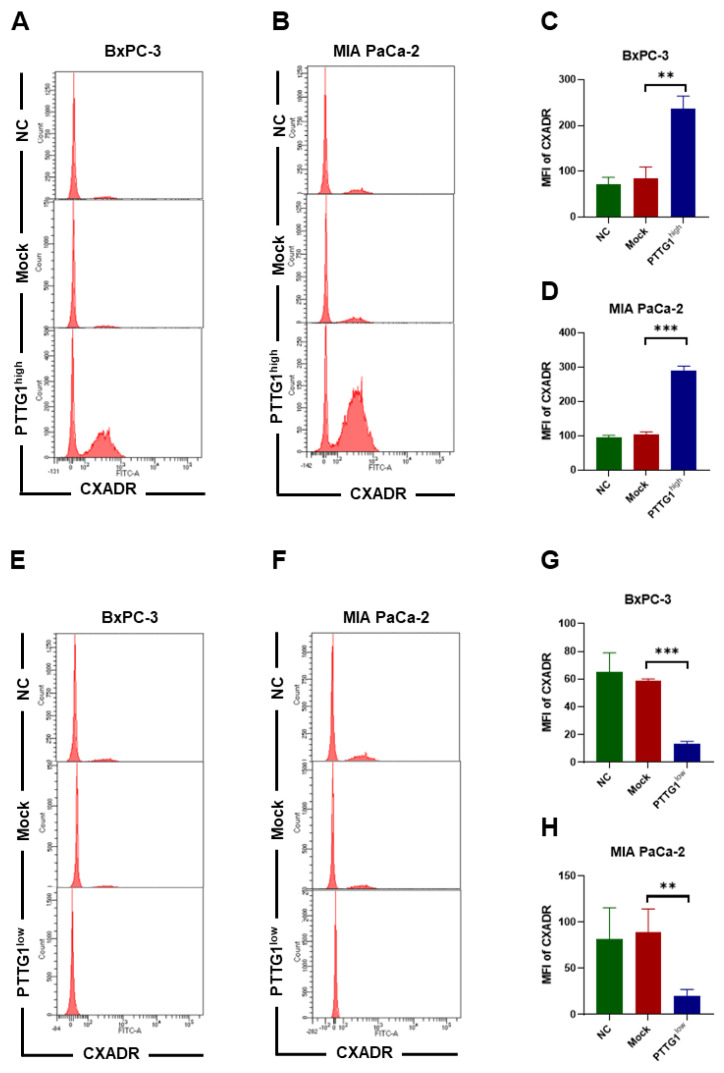
Influence of PTTG1 on CXADR expression on pancreatic cancer cells. CXADR expression on BxPC-3-PTTG1^high^ and MIA PaCa-2-PTTG1^high^ cells was detected using flow cytometry. (**A**,**B**) The representative histogram is shown. Mean fluorescence index (MFI) of CXADR expression on (**C**) BxPC-3-PTTG1^high^ and (**D**) MIA PaCa-2-PTTG1^high^ cells was analyzed. CXADR expression on BxPC-3-PTTG1^low^ and MIA PaCa-2-PTTG1^low^ cells was detected using flow cytometry. (**E**,**F**) The representative histogram is shown. MFI of CXADR expression on (**G**) BxPC-3-PTTG1^low^ and (**H**) MIA PaCa-2-PTTG1^low^ cells was analyzed. All experiments were conducted in triplicate. Nonparametric test was used to analyze differences between two groups. All data are mean ± SD. **: *p* < 0.01. ***: *p* < 0.001.

We further explore the mechanism underlying the enhancement of OAd5 entrance into cells by PTTG1. Because CXADR is the main receptor on the cell surface for OAd5 entrance into cells, we detected its expression on PTTG1 high- and low-expressed cells using flow cytometry (FCM). The results showed that CXADR expression was enhanced in BxPC-3-PTTG1^high^ (Figure 5A,C) and MIA PaCa-2-PTTG1^high^ (Figure 5B,D) cells. Furthermore, we found that CXADR expression decreased in (Figure 5E,G) BxPC-3-PTTG1^low^ and (Figure 5F,H) MIA PaCa-2-PTTG1^low^ cells. 

### 3.6. The Enhancement of Oncolytic Adenovirus Entry into Cells by PTTG1 Was Dependent on CXADR

**Figure 6:**
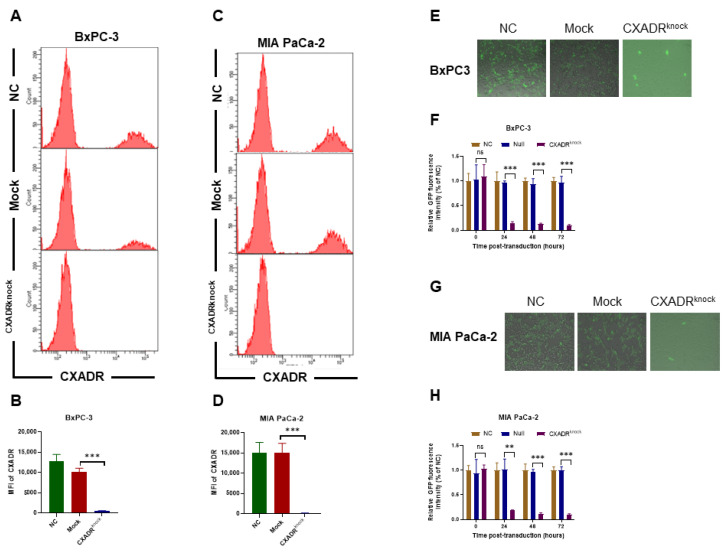
Enhancement of OAd5 entry into pancreatic cancer cells by PTTG1 was CXADR-dependent. CXADR was knocked in (**A**,**B**) BxPC-3-PTTG1^high^ and (**C**,**D**) MIA PaCa-2-PTTG1^high^ cells using CRISPR/Cas9 technology. The cells were transduced with OAd5 24 h post-transduction, and fluorescence in (**E**) BxPC-3-CXADR^low^ and (**G**) MIA PaCa-2-CXADR^low^ cells was photographed using a fluorescence microscope. Fluorescence intensity in (**F**) BxPC-3-CXADR^low^ and (**H**) MIA PaCa-2-CXADR^low^ cells were measured 0-, 24-, 48-, and 72 h post-transduction. All experiments were conducted in triplicate. Each image displayed in (**E**,**G**) is a merged bright field and fluorescent image. All experiments were conducted in triplicate. Nonparametric test was used to analyze differences between two groups. All data are mean ± SD. ns: not significant. **: *p* < 0.01. ***: *p* < 0.001.

To further investigate the role of CXADR in PTTG1 in promoting OAd5 entrance into cells, we knocked CXADR in BxPC-3-PTTG1^high^ and MIA PaCa-2-PTTG1^high^ cells using CRISPR/Cas9 technology. To validate the role of CXADR in PTTG1-mediated OAd5 entrance, we used the CXADR-knocked BxPC-3-PTTG1^high^ (Figure 6A,B) and MIA PaCa-2-PTTG1^high^ (Figure 6C,D) cells during OAd5 transduction. Then, GFP intensity assay showed that BxPC-3-PTTG1^high^ (Figure 6E,F) and MIA PaCa-2-PTTG1^high^ (Figure 6G,H) cells failed to further enhance OAd5 transduction in the presence of CXADR knockdown.

### 3.7. CXADR Expression Correlates with PTTG1 Expression in Normal and Malignant Pancreatic Tissues

**Figure 7:**
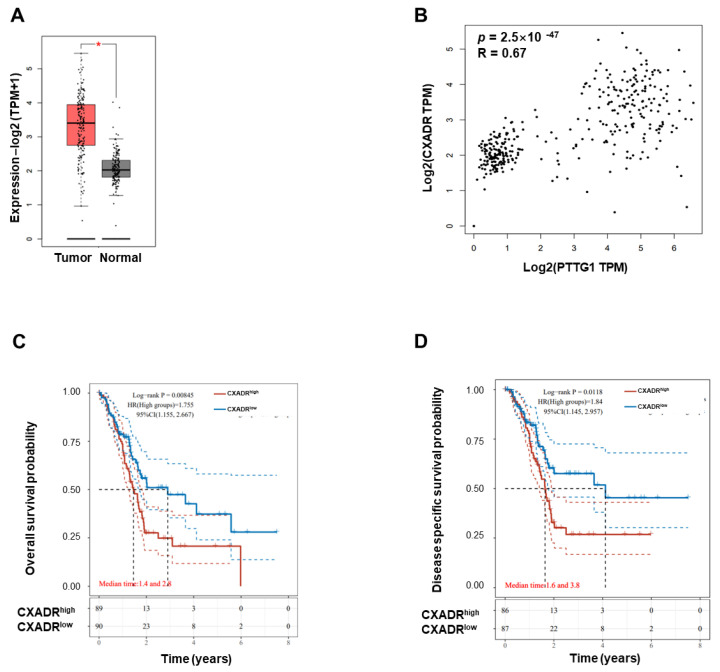
The correlation between PTTG1 and CXADR in normal and malignant pancreatic tissues. (**A**) CXADR expression analysis in PAAD from TCGA database using GEPIA 2.0. The two groups include 179 cases of pancreatic cancer tumor tissues and 171 cases of normal pancreas tissues. (**B**) The Spearman rank correlation between PTTG1 and CXADR mRNA expressions in normal and cancerous pancreatic tissues. The analysis was performed at the GEPIA 2.0 website based on the merge data from TCGA Tumor, TCGA Normal, and GTEx databases. (**C**) CXADR expression and overall survival, (**D**) PTTG1 expression and disease-specific survival were analyzed in the TCGA cohort. Hazard ratio (HR) > 1 indicates the gene is a risk factor. HR (95% Cl), the median survival time (LT50) for different groups. *: *p* < 0.05.

The in vitro experiment proved that PTTG1 could improve the expression level of CXADR on the surface of pancreatic tumor cells, so we further assessed CXADR expression in pancreatic cancer from the TCGA database. The analysis revealed that CXADR expression was higher in pancreatic cancer tumor tissues than in normal ones (Figure 7A). Then, we employed Spearman rank correlation to explore the relationship between PTTG1 and CXADR in normal and malignant pancreatic tissues. As a result, the expression of CXADR was positively correlated with PTTG1 in pancreases and pancreatic cancer tissues (Figure 7B). To assess whether the CXADR expression influenced the prognosis of pancreatic cancer, we analyzed the association between its expression and overall survival (OS), disease-free survival (DFS), progression-free survival (PFS), and disease-specific survival (DSS) in the TCGA cohort. Higher expression of CXADR was significantly associated with shorter OS (Figure 7C) and DSS (Figure 7D) in pancreatic cancer. The analysis indicates that CXADR is involved in disease progression in pancreatic cancer.

## 4. Discussion

PTTG1 is overexpressed in many tumor types, predicts poor prognosis, and is associated with therapies. For example, the blockade of PTTG1 enhances radiation-induced antitumor immunity in lung adenocarcinoma [28]. It might predict immunotherapy response in renal cell carcinoma [29], and could represent therapeutic targets in prostate medical research and clinical studies [30]. However, PTTG1 has not been extensively studied in pancreatic cancer. Our study examined PTTG1 expression levels, tumor stage, and grade using TCGA data from UCSC Xena. We found that overexpression of PTTG1 predicts a higher grade of pancreatic cancer. Additionally, we found that higher expression of PTTG1 was significantly associated with shorter OS, DFS, PFS, and DSS in pancreatic cancer. These results indicate that PTTG1 might influence disease progression in pancreatic cancer patients.

The current remedy for pancreatic cancer mainly relies on surgery and chemotherapy [31,32]. Gemcitabine [33] and 5-FU [34,35,36] are among the main chemotherapeutic choices for patients with unresectable pancreatic cancer. However, we found that IC50 of both gemcitabine and 5-FU were higher in PTTG1-overexpressed BxPC-3 and MIA PaCa-2 cells. Meanwhile, IC50 of both gemcitabine and 5-FU decreased in PTTG1 knockdown cells. Therefore, treating pancreatic cancer patients with high PTTG1 expression is difficult using gemcitabine and 5-FU. Of course, other chemotherapies might effectively treat patients with high PTTG1 expression, but this needs further exploration.

ICB-based immunotherapy is a promising cancer therapy for activating anti-tumor immune response, and it has been used in clinic for treating cancer patients [37]. We wondered whether ICBs could treat pancreatic cancer patients with high PTTG1 expression, so we analyzed the ICBs’ response with the TIDE algorithm in pancreatic cancer. It is disappointing that the ICBs’ efficiency is poor in high PTTG1 patients. Based on our research, both chemotherapy and ICBs might not be promising treatments for pancreatic cancer patients with high PTTG1 expression. Although immunotherapy for pancreatic cancer has made some progress, including ICIs, most are ineffective in pancreatic cancer patients. Therefore, potential alternatives should be explored for pancreatic cancer treatment.

Oncolytic viruses are a breakthrough in immunotherapy after ICIs. Ad is commonly used to construct oncolytic viruses due to its low pathogenicity and ease of genome modification. OAd5 is the most widely used oncolytic virus, with high transfection efficiency and the ability to generate high titer viruses effectively [38]. Oncolytic viruses could selectively propagate in and induce the lysis of malignant cells and represent an emerging new class of cancer therapeutics [39]. Numerous oncolytic viruses are under preclinical or clinical investigations, and multiple oncolytic viruses are based on genomic alterations of serotype 5 adenovirus [40,41]. Clinical trials have proved that OAd5 is safe in cancer patients and specifically eliminates tumor cells with minimized toxicity to normal cells [42].

Adenovirus type 5-based oncolytic viruses have shown potential in preclinical studies and early phase clinical trials with pancreatic cancer patients [43]. To improve tumor specificity and safety, the alterations contain a deletion of E1B55K [44,45], E3B [46], or the pRb-binding E1ACR2-region [47,48,49]. However, of course, the efficiency is poor for OAd5-transducing cancer cells that are deficient or poorly expressing adenovirus primary receptors. We infected PTTG1-overexpressed and -knocked BxPC-3 and MIA PaCa-2 cells and measured cell viability to analyze OAd5 efficiency. OAd5 efficiency was increased in PTTG1-overexpressed cells but poor in PTTG1-knocked cells. The OAd5 we used in this research could express GFP. We found that the fluorescence intensity was increased in PTTG1-overexpressed cells and decreased in PTTG1-knocked cells 24 h after OAd5 transduction. However, the magnitude of the difference in GFP intensity from the control group did not further increase over time. This is an exciting phenomenon.

At 24 h post-infection, nearly 80% of wild-type-infected cells were in S-phase [47]; we hypothesized whether PTTG1 remarkably increased OAd5 entrance into pancreatic cancer cells. Because CXADR is the primary receptor mediating OAd5 entrance into cells [50], we analyzed their expressions on PTTG1 over- and low-expressed cells. CXADR expression was enhanced in PTTG1 overexpressed BxPC-3 and MIA PaCa-2 cells and decreased in PTTG1 low-expressed cells. To further explore the role of CXADR in PTTG1-mediated OAd5 entrance, we added the CXADR inhibitor in cell supernatant during OAd5 transduction and found that PTTG1 could not enhance OAd5 transduction. Therefore, our research first showed that PTTG1-mediated OAd5 transduction was through increasing CXADR expression. Of course, our experimental results cannot completely rule out whether PTTG1 affects the replication of OAd5, and whether PTTG1 can also affect other factors that affect the entry of OAd5 into cells. Another question worth further investigation is whether PTTG1 directly increases the expression of CXADR or indirectly affects CXADR through other factors.

Using comprehensive analysis of the data of normal pancreatic tissues and pancreatic cancer tissues, we found that the expression of PTTG1 was positively correlated with CXADR. However, by analyzing the data of pancreatic cancer tissues alone, we did not find that the expression of PTTG1 was correlated with CXADR (data not shown). This might be since PTTG1 can influence CXADR at the precancerous stage, while it has a minimal impact on CXADR during the progression stage of the tumor. This also explains why the expression level of PTTG1 is higher in tumor tissue of patients in higher grades, while CXADR is not related to the grade of pancreatic cancer. This also indicates that the expression level of CXADR would not further increase with the progression of the tumor. Of course, the relationship between CXADR and prognosis is still quite close. Our partial results of CXADR for prognosis are consistent with those of PTTG1. The high expression of CXADR and PTTG1 in cancer is significantly correlated with the short OS and DSS, further demonstrating the close relationship between the two factors.

This study found that, although high expression of PTTG1 might reduce the efficacy of chemotherapy and ICBs, it was consistent with previous reports that PTTG1 could functions as a potential target in tumor therapy for various types of cancer, such as breast cancer [8], ovarian cancer [51], pituitary adenoma [52], and prostate cancer [30]. However, PTTG1 could also improve the effectiveness of OAd5. This study indicates that in clinical practice, patients can undergo puncture to obtain a very small amount of tumor tissue and detect the expression of PTTG1, indicating whether OAd5 treatment can be prioritized over chemotherapy. To further improve accuracy, CXADR expression on the surface of tumor cells can be detected to determine whether to use OAd5. This study also suggests that, in order to more accurately improve the efficacy of OAd5, a gene set that affects various therapies can be established. By detecting the expression of this gene set, it can be determined whether patients are suitable for chemotherapy, ICBs, and OAd5 therapy to achieve precision treatment.

## 5. Conclusions

In summary, PTTG1 enhances OAd5 entrance into pancreatic cancer cells through increasing CXADR expression on the cell surface. In the process of using oncolytic virus to treat pancreatic cancer, if priority is given to patients with a high expression of PTTG1 in tissues, it may provide additional benefits.

## Data Availability

Data for bioinformatics analysis was from The Cancer Genome Atlas Program (TCGA) database (https://portal.gdc.com), GEPIA 2.0 (http://gepia2.cancer-pku.cn/#index) and ACLBI database (https://www.aclbi.com/static/index.html#/).
